# Bis(2-methyl­piperidinium) naphthalene-1,5-disulfonate

**DOI:** 10.1107/S1600536812020041

**Published:** 2012-05-16

**Authors:** Qian Xu

**Affiliations:** a Ordered Matter Science Research Center, College of Chemistry and Chemical Engineering, Southeast University, Nanjing 211189, People’s Republic of China

## Abstract

In the structure of the title mol­ecular salt, 2C_6_H_14_N^+^·C_10_H_6_O_6_S_2_^2−^, the asymmetric unit consists of one 2-methyl­piperidinium cation and one-half of a naphthalene-1,5-disulfonate anion; the anion lies across a centre of symmetry. In the crystal, the cations and anions are linked through N—H⋯O hydrogen bonds, forming a two-dimensional network.

## Related literature

For general background on ferroelectric organic frameworks, see: Fu *et al.* (2009 ▶); Ye *et al.* (2006 ▶); Zhang *et al.* (2008 ▶, 2010 ▶).
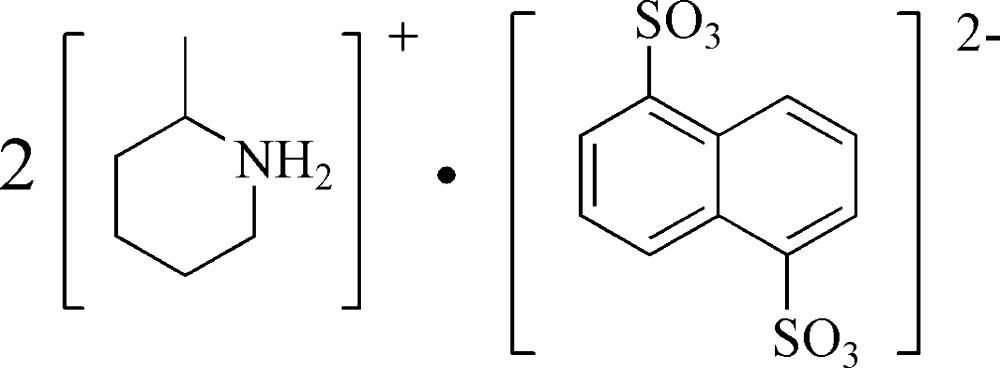


## Experimental

### 

#### Crystal data

2C_6_H_14_N^+^·C_10_H_6_O_6_S_2_^2−^
*M* *_r_* = 486.63
Monoclinic, 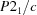

*a* = 12.040 (2) Å
*b* = 8.8133 (18) Å
*c* = 12.715 (3) Å
β = 112.62 (3)°
*V* = 1245.4 (4) Å^3^
*Z* = 2
Mo *K*α radiation
μ = 0.25 mm^−1^
*T* = 293 K
0.32 × 0.27 × 0.22 mm

#### Data collection

Rigaku SCXmini diffractometer
Absorption correction: multi-scan (*CrystalClear*; Rigaku, 2005 ▶) *T*_min_ = 0.924, *T*_max_ = 0.947
12383 measured reflections
2814 independent reflections
1942 reflections with *I* > 2σ(*I*)
*R*_int_ = 0.069
Standard reflections: ?

#### Refinement

*R*[*F* ^2^ > 2σ(*F* ^2^)] = 0.058
*wR*(*F* ^2^) = 0.134
*S* = 1.04
2814 reflections
146 parameters
H-atom parameters constrained
Δρ_max_ = 0.22 e Å^−3^
Δρ_min_ = −0.28 e Å^−3^

### 

Data collection: *CrystalClear* (Rigaku, 2005 ▶); cell refinement: *CrystalClear*; data reduction: *CrystalClear*; program(s) used to solve structure: *SHELXS97* (Sheldrick, 2008 ▶); program(s) used to refine structure: *SHELXL97* (Sheldrick, 2008 ▶); molecular graphics: *DIAMOND* (Brandenburg & Putz, 2005 ▶); software used to prepare material for publication: *SHELXL97*.

## Supplementary Material

Crystal structure: contains datablock(s) I, global. DOI: 10.1107/S1600536812020041/go2054sup1.cif

Structure factors: contains datablock(s) I. DOI: 10.1107/S1600536812020041/go2054Isup2.hkl

Supplementary material file. DOI: 10.1107/S1600536812020041/go2054Isup3.cml

Additional supplementary materials:  crystallographic information; 3D view; checkCIF report

## Figures and Tables

**Table 1:** Hydrogen-bond geometry (Å, °)

*D*—H⋯*A*	*D*—H	H⋯*A*	*D*⋯*A*	*D*—H⋯*A*
N1—H1*B*⋯O2	0.90	1.91	2.795 (3)	169
N1—H1*A*⋯O1^i^	0.90	1.93	2.820 (3)	169

Symmetry code: (i) 
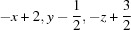
.

**Fig. 1.**
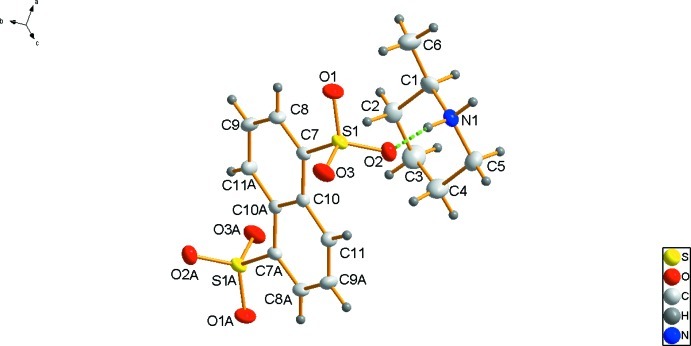
The molecular structure of the title compound, with the displacement ellipsoids drawn at the 30% probability level. Symmetry code for A: 1 - x, 1 - y, 1 - z.

**Fig. 2.**
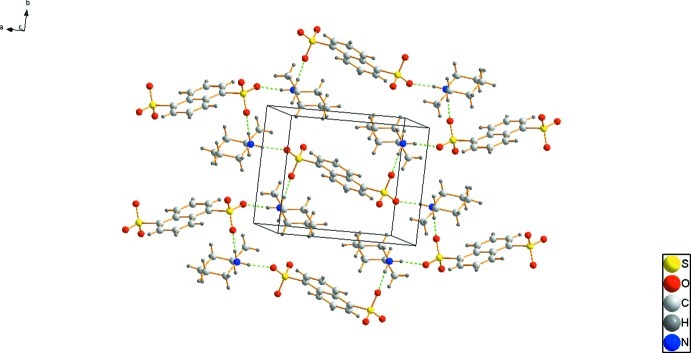
Packing diagram of the title compound, hydrogen bonds are shown as dashed lines.

## supplementary crystallographic information

### Comment

The title compound, (I), was synthesized to assess its ferroelectric properties by dielectric measurements as a function of temperature (Fu *et al.*, 2009; Ye *et al.*, 2006; Zhang *et al.*, 2008; Zhang *et al.*, 2010). In the range from 190K to near its melting point (m.p. >370K), no dielectric anomaly was observed.

A view of (I) is shown in Fig.1. Two intermolecular N–H···O hydrogen bonds form a two-dimensional network, Table, 1, Fig. 2.

### Experimental

A mixture of 2-methy piperidine (0.98 g, 10 mmol), naphthalene-1,5-disulfonic acid (2.5 g, 10 mmol) in water was stirred for several days at ambient temperature, colourless crystals were obtained.

### Refinement

Hydrogen atom positions were calculated and allowed to ride on their respective C atoms and N atoms with C–H distances of 0.93–0.98Å and N–H = 0.90Å, and with *U*_iso_(H)=1.2*U*_eq_(C or N), 1.5 *U*_iso_(C) for methyl H atoms.

### Crystal data

**Table:**

2C_6_H_14_N^+^·C_10_H_6_O_6_S_2_^2^^−^	*F*(000) = 520
*M**_r_* = 486.63	*D*_x_ = 1.298 Mg m^−^^3^
Monoclinic, *P*2_1_/*c*	Mo *K*α radiation, λ = 0.71073 Å
Hall symbol: -P 2ybc	Cell parameters from 2814 reflections
*a* = 12.040 (2) Å	θ = 3.1–27.5°
*b* = 8.8133 (18) Å	µ = 0.25 mm^−^^1^
*c* = 12.715 (3) Å	*T* = 293 K
β = 112.62 (3)°	Block, colourless
*V* = 1245.4 (4) Å^3^	0.32 × 0.27 × 0.22 mm
*Z* = 2	

### Data collection

**Table:**

Rigaku SCXmini diffractometer	2814 independent reflections
Radiation source: fine-focus sealed tube	1942 reflections with *I* > 2σ(*I*)
Graphite monochromator	*R*_int_ = 0.069
CCD_Profile_fitting scans	θ_max_ = 27.5°, θ_min_ = 3.0°
Absorption correction: multi-scan (*CrystalClear*; Rigaku, 2005)	*h* = −15→15
*T*_min_ = 0.924, *T*_max_ = 0.947	*k* = −11→11
12383 measured reflections	*l* = −16→16

### Refinement

**Table:**

Refinement on *F*^2^	Primary atom site location: structure-invariant direct methods
Least-squares matrix: full	Secondary atom site location: difference Fourier map
*R*[*F*^2^ > 2σ(*F*^2^)] = 0.058	Hydrogen site location: inferred from neighbouring sites
*wR*(*F*^2^) = 0.134	H-atom parameters constrained
*S* = 1.04	*w* = 1/[σ^2^(*F*_o_^2^) + (0.0546*P*)^2^ + 0.481*P*] where *P* = (*F*_o_^2^ + 2*F*_c_^2^)/3
2814 reflections	(Δ/σ)_max_ < 0.001
146 parameters	Δρ_max_ = 0.22 e Å^−^^3^
0 restraints	Δρ_min_ = −0.28 e Å^−^^3^

### Special details

**Table:**

Geometry. All e.s.d.'s (except the e.s.d. in the dihedral angle between two l.s. planes) are estimated using the full covariance matrix. The cell e.s.d.'s are taken into account individually in the estimation of e.s.d.'s in distances, angles and torsion angles; correlations between e.s.d.'s in cell parameters are only used when they are defined by crystal symmetry. An approximate (isotropic) treatment of cell e.s.d.'s is used for estimating e.s.d.'s involving l.s. planes.
Refinement. Refinement of *F*^2^ against ALL reflections. The weighted *R*-factor *wR* and goodness of fit *S* are based on *F*^2^, conventional *R*-factors *R* are based on *F*, with *F* set to zero for negative *F*^2^. The threshold expression of *F*^2^ > σ(*F*^2^) is used only for calculating *R*-factors(gt) *etc*. and is not relevant to the choice of reflections for refinement. *R*-factors based on *F*^2^ are statistically about twice as large as those based on *F*, and *R*- factors based on ALL data will be even larger.

### Fractional atomic coordinates and isotropic or equivalent isotropic displacement parameters (Å^2^)

**Table:**

	*x*	*y*	*z*	*U*_iso_*/*U*_eq_	
S1	0.78457 (5)	0.59654 (7)	0.69286 (5)	0.0339 (2)	
O1	0.88140 (15)	0.6629 (2)	0.66486 (16)	0.0499 (5)	
O2	0.81475 (16)	0.4419 (2)	0.73778 (15)	0.0452 (5)	
O3	0.74714 (16)	0.6921 (2)	0.76559 (16)	0.0508 (5)	
C7	0.6587 (2)	0.5777 (3)	0.5596 (2)	0.0306 (5)	
C8	0.6710 (2)	0.6257 (3)	0.4618 (2)	0.0376 (6)	
H8	0.7426	0.6706	0.4663	0.045*	
C9	0.5757 (2)	0.6074 (3)	0.3545 (2)	0.0439 (7)	
H9	0.5848	0.6414	0.2890	0.053*	
C10	0.5479 (2)	0.5101 (3)	0.55522 (19)	0.0286 (5)	
C11	0.5297 (2)	0.4597 (3)	0.6538 (2)	0.0388 (6)	
H11	0.5907	0.4727	0.7253	0.047*	
N1	0.88441 (18)	0.1722 (2)	0.66473 (18)	0.0395 (5)	
H1A	0.9617	0.1614	0.7127	0.047*	
H1B	0.8586	0.2628	0.6790	0.047*	
C1	0.8782 (2)	0.1714 (3)	0.5439 (2)	0.0475 (7)	
H1	0.9076	0.0732	0.5292	0.057*	
C2	0.7463 (3)	0.1887 (3)	0.4635 (2)	0.0509 (8)	
H2A	0.7176	0.2877	0.4750	0.061*	
H2B	0.7404	0.1834	0.3854	0.061*	
C3	0.6666 (3)	0.0666 (4)	0.4827 (3)	0.0713 (10)	
H3A	0.6888	−0.0317	0.4624	0.086*	
H3B	0.5833	0.0860	0.4341	0.086*	
C4	0.6799 (3)	0.0642 (4)	0.6077 (3)	0.0668 (10)	
H4A	0.6468	0.1569	0.6248	0.080*	
H4B	0.6345	−0.0204	0.6196	0.080*	
C5	0.8109 (3)	0.0494 (3)	0.6881 (3)	0.0552 (8)	
H5A	0.8172	0.0568	0.7663	0.066*	
H5B	0.8415	−0.0490	0.6780	0.066*	
C6	0.9591 (3)	0.2953 (4)	0.5302 (3)	0.0726 (10)	
H6A	0.9276	0.3927	0.5383	0.109*	
H6B	0.9623	0.2879	0.4561	0.109*	
H6C	1.0387	0.2838	0.5875	0.109*	

### Atomic displacement parameters (Å^2^)

**Table:**

	*U*^11^	*U*^22^	*U*^33^	*U*^12^	*U*^13^	*U*^23^
S1	0.0244 (3)	0.0376 (4)	0.0378 (4)	−0.0008 (3)	0.0099 (2)	−0.0067 (3)
O1	0.0283 (9)	0.0686 (14)	0.0508 (12)	−0.0130 (9)	0.0130 (8)	−0.0050 (10)
O2	0.0429 (11)	0.0441 (11)	0.0426 (11)	0.0092 (8)	0.0099 (8)	0.0024 (8)
O3	0.0425 (11)	0.0563 (13)	0.0549 (12)	−0.0018 (9)	0.0201 (9)	−0.0229 (10)
C7	0.0239 (12)	0.0284 (12)	0.0400 (14)	−0.0010 (10)	0.0128 (10)	0.0001 (10)
C8	0.0287 (13)	0.0395 (15)	0.0466 (16)	−0.0077 (11)	0.0166 (12)	0.0011 (12)
C9	0.0392 (14)	0.0593 (18)	0.0380 (15)	−0.0038 (13)	0.0200 (12)	0.0103 (13)
C10	0.0260 (12)	0.0274 (12)	0.0318 (13)	0.0005 (10)	0.0103 (9)	0.0013 (10)
C11	0.0304 (13)	0.0516 (16)	0.0307 (14)	−0.0030 (12)	0.0077 (11)	0.0052 (11)
N1	0.0324 (11)	0.0387 (13)	0.0425 (13)	0.0040 (9)	0.0088 (10)	0.0010 (10)
C1	0.0460 (16)	0.0509 (18)	0.0490 (17)	0.0072 (13)	0.0221 (14)	−0.0056 (13)
C2	0.0547 (18)	0.0561 (19)	0.0350 (16)	−0.0007 (15)	0.0097 (13)	−0.0042 (13)
C3	0.061 (2)	0.074 (2)	0.061 (2)	−0.0227 (18)	0.0029 (17)	−0.0099 (18)
C4	0.0486 (19)	0.075 (2)	0.070 (2)	−0.0180 (17)	0.0150 (17)	0.0046 (17)
C5	0.0578 (19)	0.0477 (18)	0.0576 (19)	−0.0046 (14)	0.0196 (16)	0.0110 (14)
C6	0.055 (2)	0.102 (3)	0.064 (2)	−0.0095 (19)	0.0280 (17)	0.017 (2)

### Geometric parameters (Å, º)

**Table:**

S1—O3	1.4452 (18)	C1—C6	1.518 (4)
S1—O1	1.4652 (18)	C1—C2	1.530 (4)
S1—O2	1.4687 (19)	C1—H1	0.9800
S1—C7	1.794 (3)	C2—C3	1.523 (4)
C7—C8	1.374 (3)	C2—H2A	0.9700
C7—C10	1.442 (3)	C2—H2B	0.9700
C8—C9	1.414 (4)	C3—C4	1.535 (5)
C8—H8	0.9300	C3—H3A	0.9700
C9—C11^i^	1.367 (3)	C3—H3B	0.9700
C9—H9	0.9300	C4—C5	1.519 (4)
C10—C11	1.424 (3)	C4—H4A	0.9700
C10—C10^i^	1.445 (4)	C4—H4B	0.9700
C11—C9^i^	1.367 (3)	C5—H5A	0.9700
C11—H11	0.9300	C5—H5B	0.9700
N1—C5	1.498 (3)	C6—H6A	0.9600
N1—C1	1.509 (3)	C6—H6B	0.9600
N1—H1A	0.90	C6—H6C	0.9600
N1—H1B	0.90		
			
O3—S1—O1	113.33 (12)	C2—C1—H1	108.6
O3—S1—O2	112.56 (12)	C3—C2—C1	112.2 (2)
O1—S1—O2	111.33 (12)	C3—C2—H2A	109.2
O3—S1—C7	107.48 (11)	C1—C2—H2A	109.2
O1—S1—C7	105.63 (11)	C3—C2—H2B	109.2
O2—S1—C7	105.90 (11)	C1—C2—H2B	109.2
C8—C7—C10	120.8 (2)	H2A—C2—H2B	107.9
C8—C7—S1	118.69 (18)	C2—C3—C4	110.8 (2)
C10—C7—S1	120.49 (18)	C2—C3—H3A	109.5
C7—C8—C9	120.6 (2)	C4—C3—H3A	109.5
C7—C8—H8	119.7	C2—C3—H3B	109.5
C9—C8—H8	119.7	C4—C3—H3B	109.5
C11^i^—C9—C8	120.6 (2)	H3A—C3—H3B	108.1
C11^i^—C9—H9	119.7	C5—C4—C3	111.5 (3)
C8—C9—H9	119.7	C5—C4—H4A	109.3
C11—C10—C7	123.2 (2)	C3—C4—H4A	109.3
C11—C10—C10^i^	118.8 (3)	C5—C4—H4B	109.3
C7—C10—C10^i^	118.0 (3)	C3—C4—H4B	109.3
C9^i^—C11—C10	121.3 (2)	H4A—C4—H4B	108.0
C9^i^—C11—H11	119.3	N1—C5—C4	110.1 (2)
C10—C11—H11	119.3	N1—C5—H5A	109.6
C5—N1—C1	113.4 (2)	C4—C5—H5A	109.6
C5—N1—H1A	109.0	N1—C5—H5B	109.6
C1—N1—H1A	108.8	C4—C5—H5B	109.6
C5—N1—H1B	108.9	H5A—C5—H5B	108.1
C1—N1—H1B	108.9	C1—C6—H6A	109.5
H1A—N1—H1B	107.7	C1—C6—H6B	109.5
N1—C1—C6	109.3 (2)	H6A—C6—H6B	109.5
N1—C1—C2	108.2 (2)	C1—C6—H6C	109.5
C6—C1—C2	113.5 (3)	H6A—C6—H6C	109.5
N1—C1—H1	108.6	H6B—C6—H6C	109.5
C6—C1—H1	108.6		
			
O3—S1—C7—C8	120.4 (2)	S1—C7—C10—C10^i^	−176.5 (2)
O1—S1—C7—C8	−0.8 (2)	C7—C10—C11—C9^i^	−178.3 (2)
O2—S1—C7—C8	−119.0 (2)	C10^i^—C10—C11—C9^i^	0.4 (4)
O3—S1—C7—C10	−61.0 (2)	C5—N1—C1—C6	−177.8 (2)
O1—S1—C7—C10	177.70 (18)	C5—N1—C1—C2	58.2 (3)
O2—S1—C7—C10	59.5 (2)	N1—C1—C2—C3	−56.1 (3)
C10—C7—C8—C9	−1.1 (4)	C6—C1—C2—C3	−177.6 (3)
S1—C7—C8—C9	177.49 (19)	C1—C2—C3—C4	55.1 (4)
C7—C8—C9—C11^i^	−0.7 (4)	C2—C3—C4—C5	−53.6 (4)
C8—C7—C10—C11	−179.3 (2)	C1—N1—C5—C4	−58.2 (3)
S1—C7—C10—C11	2.1 (3)	C3—C4—C5—N1	54.5 (4)
C8—C7—C10—C10^i^	2.0 (4)		

Symmetry code: (i) −*x*+1, −*y*+1, −*z*+1.

### Hydrogen-bond geometry (Å, º)

**Table:**

*D*—H···*A*	*D*—H	H···*A*	*D*···*A*	*D*—H···*A*
N1—H1*B*···O2	0.90	1.91	2.795 (3)	169
N1—H1*A*···O1^ii^	0.90	1.93	2.820 (3)	169

Symmetry code: (ii) −*x*+2, *y*−1/2, −*z*+3/2.

## References

[bb1] Brandenburg, K. & Putz, H. (2005). *DIAMOND* Crystal Impact GbR, Bonn, Germany.

[bb2] Fu, D.-W., Ge, J.-Z., Dai, J., Ye, H.-Y. & Qu, Z.-R. (2009). *Inorg. Chem. Commun.* **12**, 994–997.

[bb3] Rigaku (2005). *CrystalClear* Rigaku Corporation, Tokyo, Japan.

[bb4] Sheldrick, G. M. (2008). *Acta Cryst.* A**64**, 112–122. 10.1107/S0108767307043930 18156677

[bb5] Ye, Q., Song, Y.-M., Wang, G.-X., Chen, K. & Fu, D.-W. (2006). *J. Am. Chem. Soc.* **128**, 6554–6555. 10.1021/ja060856p 16704244

[bb6] Zhang, W., Xiong, R.-G. & Huang, S.-P. D. (2008). *J. Am. Chem. Soc.* **130**, 10468–10469. 10.1021/ja803021v 18636707

[bb7] Zhang, W., Ye, H.-Y., Cai, H.-L., Ge, J.-Z. & Xiong, R.-G. (2010). *J. Am. Chem. Soc.* **132**, 7300–7302. 10.1021/ja102573h 20459097

